# Infantile Cortical Hyperostosis: Report of a Case with Observations on Clinical Manifestations, Radiology, and Pathology with a Late Follow-Up of Eight Years

**DOI:** 10.1155/2016/2073854

**Published:** 2016-12-06

**Authors:** Pedro Carlos M. Sarmento Pinheiro, Ierecê Lins Aymore, Armando Rocha Amoedo, Paulo Miguel Hemais

**Affiliations:** ^1^Orthopedic Department, Jesus Children's Hospital, Rio de Janeiro, RJ, Brazil; ^2^Oncological and Pathological Department of National Institute of Orthopedic and Traumatology (INTO), Rio de Janeiro, RJ, Brazil; ^3^Radiological Department, Jesus Children's Hospital, Rio de Janeiro, RJ, Brazil; ^4^Department of Radiology, National Institute of Orthopedic and Traumatology (INTO), Rio de Janeiro, RJ, Brazil

## Abstract

*Purpose*. The purpose of our study was to investigate clinical manifestations, roentgen images, histopathological studies, and evolution of the disease in patient displaying infantile cortical hyperostosis.* Methods*. Roentgenograms were made to evaluate a neonatal patient presenting multiple soft-tissue swellings. The initial radiographs insinuated that the disease had been present for some time* in utero*. Bone puncture biopsy of the tibia for histopathological observation and diagnosis conclusions was performed.* Results*. The disease was demonstrated radiographically by massive cortical diaphyseal thickening and also extensive periosteal new bone formation surrounding several bones. Results in blood count were as follows: discrete anemia, moderate leukocytosis, and elevated sedimentation rate. Histological pattern of tissue removed from tibia showed lamellar cortical bones and hyperplasia. Biopsy studies disclosed no evidence of neoplasia as well as of bacterial infection.* Comments*. Clinical manifestations in a neonatal patient displaying infantile cortical hyperostosis have gradually decreased. Radiograph findings have demonstrated complete recovery of bones manifested by the disease. The pathologic findings are in accordance with previous microscopic examination summarized by the literature. Total patient cure, without sequels, could be demonstrated.

## 1. Introduction

This report is a description of a patient who presented infantile cortical hyperostosis (ICH), also called Caffey's disease. The condition is a self-limited entity of undetermined cause [1]. In the literature, authors have hypothesized severe immunologic defects and a viral cause as possible predisposing factors [2]. The disease affects males and females in the same proportion [1]. After birth clinical aspects of the disease can be observed during the neonatal phase: tender soft-tissue swellings, hyperirritability, and fever. Roentgen finding manifestations have shown subperiosteal new bone formation, chiefly involving diaphysis of long bones, and thickened mandible. Microscopic pathology for histological pattern study was performed. These aspects associated with longer duration of the disease definitely represent a rare syndrome described by John Caffey and William A. Silverman in July 1945. Despite being well identified, much is still to be understood about this intriguing disease [1].

## 2. History

There was no record of description, of either radiographic or clinical aspects of this disease, until Röske (1930) [3] described an individual case similar to ICH, which he could not classify.

Caffey (1939) [4] observed bone changes in a nonsyphilitic infant.

de Toni (1943) [5] early recognized a single case of congenital and regressive aspects of ICH in an Italian infant.

Caffey and Silverman (1945) [6] described four patients who combined clinical and roentgen findings, appearing to constitute new infantile nonsyphilitic syndrome named infantile cortical hyperostosis, as a distinct disease.

Caffey (1946) [7] published six additional patients displaying ICH characteristic findings.

Zeben Van (1948) [8] reported the affection in a family of two brothers and one cousin.

Macgregor and Davies (1949) [9] collected twenty-eight cases by various authors, and clinical details were tabulated.

Bennett and Nelson (1953) [10] studied a dead fetus with ICH.

Jackson and Lyne (1979) [11] mentioned in detail many complications associated with ICH. Pazzaglia et al. (1985) [12] reported a postmortem study in which many bones were examined by light and electron microscopy.

 Agrawal et al. (2011) [13] presented a bone scan done with 99m Tc-MDP and fluorodeoxy glucose positron emission tomography (FDG PET), which have shown diffusely increased tracer uptake and patchy FDG in the long bones.

## 3. Case Presentation

**Figure 1:**
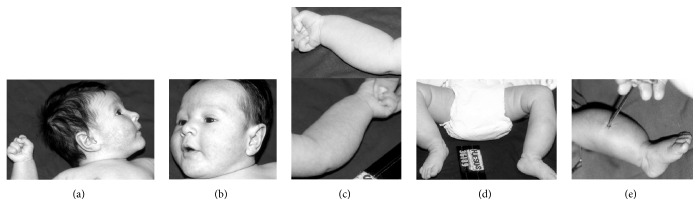
Clinical manifestations. ((a) and (b)) Facial swelling of ICH neonatal patient at 17 days of age. The swelling of the right and left cheeks is evident. (c) Photograph of the right and left forearms at 17 days of age shows that they are diffusely swollen. (d) Photograph of the lower limbs showing both legs visibly swollen. (e) Bone biopsy when patient was two months of age from the middle shaft of the tibia.

Typology of the design employed in this study was observational, interventional, and longitudinal (follow-up) with retrospective characteristics. This report is a narrative of clinical, roentgen, and pathologic findings in a 17-day-old white boy (B. J. P. M.), weighing 3.950 kilograms, who was born in Rio de Janeiro, Brazil, on July 22, 2000. The patient, a male, was referred to our service for investigation, diagnosis, and treatment. The child was product of a normal gestation by caesarean section and was born apparently normal and healthy. Parents were not close relatives (consanguineous). At the admission, the mother's clinical chief complaint was irritability, facial swelling (Figures 1(a) and 1(b)), and pain when the patient was handled. In the physical examination, the patient presented involvement of both sides of his face with enlarged bones of mandible, forearm, legs, and clavicles. Ventral bowing of tibias during the first days of life was also observed. Both clavicles were thickened upon palpation. Upper and lower limbs with multiple soft-tissues (Figures 1(c) and 1(d)) and tender swellings were noted. Irritable patient with range-of-motion joints limitations was detected. There was no pyrexia during presentation. Abdominal and pelvic structures were normal. No clinical evidence of hemorrhage was found during follow-up on disease-affected patient. Regional lymph nodes were not enlarged. Major portions of the massive swellings were located in the cheeks, forearms, and legs. There were pain and tenderness during active and passive forearm and leg manipulations. Multiple skeletal thickenings and periosteal new bone formation surrounding long bone diaphyses as well as cortical thickening in mandible in the first X-ray examinations were recognized. Bone puncture biopsy of tibia was performed to evaluate and observe histological pattern (Figure 1(e)). Pathological studies were summarized as follows: thickening of periosteum, diffuse diaphyseal remodeling of affected bones and absence of cortical bone, intense proliferation of subperiosteal cells and new bone formation, and fibrosis of bone marrow were observed.

## 4. Results

### 4.1. Laboratory Findings

Results in blood count were as follows: red cells 3,730,000 per cubic millimeter (c.mm.), discrete anemia; white blood cells 15,300 per (c.mm.), moderate leukocytosis; and sedimentation rate of 35 mm at the end of one hour, which was elevated. Hemoglobin was 11.3 gm% and hematocrit 32%. In the blood serum, the alkaline phosphatase value was in abnormal quantities (243 U/L). Plaques were 455.00/mm^3^. White cells, basophils 1%; segmented 62%; eosinophils 1%; monocytes 7%; and lymphocytes, 29% were observed. Blood serum contained normal quantities of calcium and phosphorus levels. Abnormalities in urinary findings as hematuria were not evident.

### 4.2. Roentgenographic Findings

**Figure 2:**
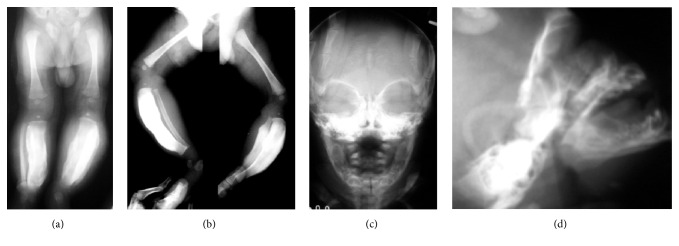
Radiographs of the cortical hyperostosis. ((a) and (b)) Massive cortical thickening and bowing of both tibias are shown. Thickening of left fibula is observed. Soft parts of both legs are thickened in frontal and lateral view. ((c) and (d)) Massive external cortical of mandible in frontal and lateral projection. Mandible is swollen.

**Figure 3:**
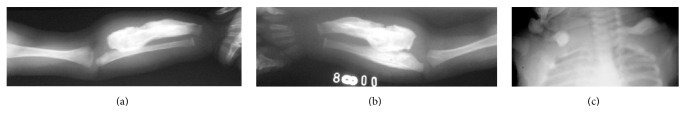
Massive cortical thickening of bones. ((a) and (b)) Right and left forearms are diffusely swollen. Massive irregular cortical thickening is visible in both radiuses and left ulna. All the bones beneath swellings show massive hyperostosis. (c) Thick sclerotic hyperostosis surrounding the clavicles is noted.

**Figure 4:**
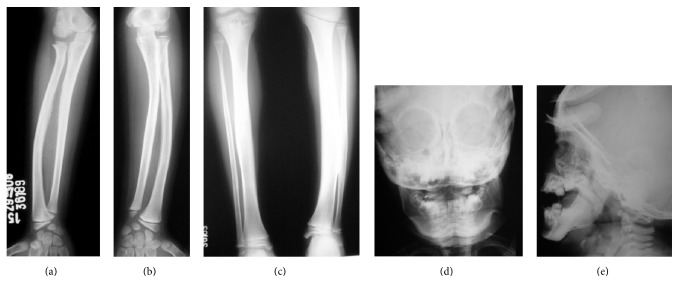
Radiographs showing spontaneous resolution. ((a) and (b)) Radiograph findings of right and left forearms were normal in follow-up examination at eight years of age. (c) The roentgenographic findings of tibias and left fibula were normal in follow-up examination at eight years of age. ((d) and (e)) Swelling of mandible had diminished and disappeared completely at 1 + 2 years of age (frontal and lateral projection).

Roentgenographic examination of the skeleton called attention to the presence of the disease studied. Scattered hyperostosis (massive cortical diaphyseal thickening) and extensive periosteal new bone formation surrounding several bones were observed. Both tibias and the left fibula (Figures 2(a) and 2(b)), the mandible (Figures 2(c) and 2(d)), both radiuses (Figures 3(a) and 3(b)), left ulna (Figure 3(b)), and the clavicles (Figure 3(c)) had been affected with massive hyperostosis. The right ulna and fibula bones were unaffected. Growth plates and epiphysis were normal in appearance, without alterations. Radiuses, tibias, clavicles, and mandible were the predominant bones to be modified. General outlook was for bone spontaneous resolution [12] (Figures 4(a), 4(b), and 4(c)).

### 4.3. Pathologic Findings

**Figure 5:**
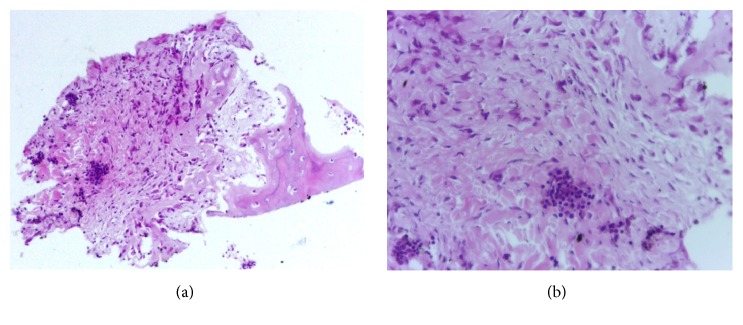
Histological study. (a) Periosteal reaction with inflammatory grouped lymphocytes infiltrate. (b) Detail of the fibroblastic periosteal proliferation with osteoid formation and lymphocytic infiltrate.

Bone biopsy for microscopic examination of the middle shaft of tibia was performed when patient was two months of age. Needle puncture for histopathological studies was done. Histological pattern of tissue removed from tibia showed lamellar cortical bones and hyperplasia. Evidences of inflammation, subperiosteal hemorrhage, and neoplasia were excluded. In our study, biopsy studies disclosed no evidence of neoplasia as well as of bacterial infection (Figure 5).

## 5. Discussion

Several reports mention the following:* osteogenesis imperfecta*, inflammatory pathogenesis, metabolic causes, syphilis, scurvy, infection, trauma, child abuse, obstetrical traumas, Kenny-Caffey disease, and neoplasia as differential diagnosis [7, 14–17]. Etiology of infantile cortical hyperostosis has not yet been established. The etiology is unknown. Cause and pathogenesis of the disorder remain undetermined and obscure [7]. Viral exogenous agents have to be on top of the possibility list as well as immunologic defect predisposition [2]. Severe immunologic defect, bacterial infection, allergy, genetic transmission, and collagen disease must be among the possibilities for the cause of this disease [2, 7]. The literature shows that first manifestations can be observed during the first five months of age, early infancy [12]. In our patient, diagnosis was made in neonatal period. Usually primary complaint is appearance of tender swellings. Authors have observed that the clinical course is highly variable, with eventual recovery [6, 12, 14, 15]. Patient's health was followed up monthly and showed no complication until he regained a normal life. Standing and walking are usually delayed [7, 14, 18]. Beginning of regular gait in our patient started when he was two years old. Mandible involvement in the literature is about 75–80% [13]. Changes in mandible comprise one of the important signals in ICH and were present in our patient. Massive hyperostosis of the mandible healed when patient was 1 + 2 years of age. Laboratory analyses of blood count, white blood cells, sedimentation rate, hematocrit, and serum alkaline phosphatase were documented. Results found in our patient were in accordance with the values related in literature. Diagnosis is based on clinical findings and X-ray reactions in certain bones. The most frequently affected bones in the literature are as follows: mandible, ribs, and the clavicles [6, 7, 19]. Bones with less pronounced changes are as follows: humeri, ulnas, ribs, femurs, scapulas, and fibulas [6, 7]. The most affected bones in this work are found to be in accordance with the ones published in the literature. Knowledge of pathology findings from biopsy can also be noted [7, 12, 20, 21]. Our histological pathological study matched modifications related in literature. Disease components, that is, soft-tissue swellings and cortical hyperostosis in neighboring bones, were in agreement with our patient [6, 7]. Treatment was generally symptomatic [22]. Use of analgesics on the patient up to seven months of age was enough to control the painful symptomatology. Prognosis was good [15, 23]. We would recommend a genetic screening not only to confirm the diagnosis of Caffey disease but also to provide genetic counseling for future generations. Findings obtained in this work and the course of the disease have represented a classical ICH type.

## 6. Comments

Clinical manifestations have shown us that soft-tissue swellings of patient displaying ICH have gradually decreased. Radiograph findings have shown complete recovery of bones manifested by the disease. The described irregular pathologic findings are in accordance with previous microscopic examinations summarized by the literature. After eight years of evolution, total patient cure, without sequels, could be demonstrated.
